# A 6-Month Evaluation of the Peer-Ceived Momentary Assessment Method in a Small Sample of Liver Transplant Patients and Their Support Persons: Longitudinal Observational Study

**DOI:** 10.2196/55907

**Published:** 2025-11-27

**Authors:** Allan Berrocal, Waldo Concepcion, Katarzyna Wac

**Affiliations:** 1 Quality of Life Technologies Lab Center for Informatics University of Geneva Geneva Switzerland; 2 Mohammed Bin Rashid University of Medicine and Health Sciences Dubai, null United Arab Emirates

**Keywords:** peer-ceived momentary assessment, PeerMA, ecological momentary assessment, EMA, human state assessment, behavior modeling, human-smartphone interaction, digital health, well-being, mobile phone

## Abstract

**Background:**

Patient-reported outcomes, including ecological momentary assessments (EMAs), are acquired from patients via repeated self-reports of their perceived momentary physical and emotional states before and after medical procedures. Patient-reported outcomes are used to measure health outcomes and quality of care. However, certain observable states or behaviors (eg, moods such as fatigue, hope, or medication adherence), or behaviors suggestive of health decline (eg, depression, cognitive decline), are not easily measured via self-reports in certain situations (eg, patients undergoing certain medical procedures, patients with dementia, and others). The peer-ceived momentary assessment (PeerMA) method involves support persons or peers (eg, family members and friends) to report their perception of a patient’s subjective physical and emotional states and has been validated in healthy populations.

**Objective:**

We examined the value of the PeerMA method in assessing the disease progression and recovery pathways of patients undergoing liver transplantation. Herein, the PeerMA method is operationalized via the patient’s informal caregivers and the patient-based EMA, and wearable-based physical activity datasets from the patients. We report the feasibility results and human factors influencing the acceptance and reliability of the PeerMA method in a small study comprising 8 patients and support persons.

**Methods:**

We conducted a longitudinal observational study of 6 months (autumn 2019 to spring 2020), collecting EMA/self-reports from 8 patients (at the liver transplant clinic at Stanford University Hospital, California) about their perceived levels of hope, sleep, fatigue, depression, and pain in addition to PeerMA-based reports of the same aspects from 7 caregivers. We collected physical activity records from 5 patients using a Fitbit bracelet. Participants completed pre- and poststudy surveys, contributing qualitative data. We implemented the PeerMA method using a smartphone app, making it easy to use by both patients and support persons.

**Results:**

We collected 1142 patient-days and 976 support person–days. On average, each patient received 103 EMAs and responded to 64 (63%) of them, while support persons received 87 PeerMAs and responded to 64 (74%) of them. We report empirical evidence about the methodological feasibility of PeerMA, showing its dual and unique information streams unavailable by EMA alone. We show examples where support person assessments and physical activity data can inform health professionals about the actual state of a patient regarding outcomes such as hope, sleep quality, fatigue, pain, and depression. We discuss human factors influencing the acceptance of the method and make methodological recommendations.

**Conclusions:**

It is possible to leverage data acquired via the PeerMA method and a wearable activity monitor to complement EMA. The PeerMA method incorporates frequent observations from support persons in patients’ daily lives, which can be compared and analyzed next to the patient’s self-reports. Such data may help to study and assist patients during disease recovery, which is beneficial for patients recovering from an organ transplant.

## Introduction

### Background

The ecological momentary assessment (EMA) method was introduced by Stone and Shiffman [1] as a technique to repeatedly collect individual momentary self-assessments for a particular outcome of interest (eg, mood, pain, and happiness), which could be combined with other digital data streams predominantly obtained from smartphone sensors and wearables. An extension of this method, known as peer-ceived momentary assessment (PeerMA), was proposed by Berrocal and Wac [2] and involves peers of the individual (eg, friends or family members) to report peer-based momentary assessments about how they perceive the individual’s internal states and external observable behaviors. PeerMA is inspired by the concept of observer and EMA’s theoretical and methodological principles. The PeerMA method is a form of EMA where the report is completed by a designated peer of an individual, in the same time observation window as when the individual is prompted to complete his/her own EMA. Peers are defined as close, trusted friends or family members taking the role of observers of the individual completing the EMA. So far, the PeerMA method has been used only in studies involving healthy participants [3]; consequently, the value of PeerMA in patient populations is still an open area of research to which this work contributes.

### Related Work

The EMA method has been used in psychology to assess human aspects such as emotional awareness [4], depression [5], happiness [6], or human virtues [7]. In clinical settings [8], EMA was used to assess human states and behavioral disorders such as mood dysregulation, anxiety, substance use, or psychosis [9]. EMA was also used to assess mood and stress changes [10] in patients with chronic conditions such as fatigue, acquired immunodeficiency syndrome, migraine, breast cancer, or kidney disease. Besides health-related uses, the EMA method has been applied to organizational research [11] and in computer science, particularly in the subfields of ubiquitous computing and human-computer interaction [12].

In the literature on health care, the concept of peer is also known as proxy or observer [13] or informant [14]. This concept of peers has been used in multiple areas, including behavior and personality assessment [14-18], chronic stress detection [19], and mental health [20]. In clinical settings, proxies often assess a patient’s condition when the patient cannot express himself or herself. For instance, children or patients with dementia [13] or when the patient cannot complete a self-report due to other factors [21]. In clinical care, proxies have been used for rehabilitation and general health [21-24], cancer [25-27], multiple sclerosis, and other chronic diseases [28], among others. Observers can provide assessments about a patient’s condition in addition to the patient’s self-report. In both clinical and nonclinical scenarios, observers or peers have been documented primarily in studies involving one-time assessments or in longitudinal studies with test and retest periods separated by months or years [29]. In the PeerMA method, peers (observers or support persons) who are usually informal caregivers (eg, friends or family members) contribute more frequently to the patient’s care, for example, multiple times per day, which may be important in certain patients. That can be especially important in high-risk populations such as patients undergoing organ transplants. That relationship between the patient and his/her support person could be understood as a form of social support, an important element influencing the patient’s quality of life during and after the recovery period [30-33]. Such social support begins with the closest circles, like family and caregivers. Overall, besides complementing someone’s self-assessments, peers can also be allies for an individual recovering from an addiction (eg, eating disorders, smoking, and gambling). In this case, peers’ assessments could help prevent relapses and their negative consequences.

The work presented in this paper makes a unique contribution by exploring the use of the PeerMA method [2], implying an acquisition of a patient’s support persons’ assessment to complement the patients’ self-reported information obtained via EMA and wearable activity monitors. The combination of these patients’ data streams is promising for care personnel to learn more about disease progression and recovery pathways of patients undergoing a liver transplant. This research has foreseeable implications for supporting person-assisted health management programs, ubiquitous technologies, and mobile human-computer interaction.

### Study Aims

In this study, we aimed to explore the following questions:

- Aim 1: Is it feasible to leverage the PeerMA method in conjunction with EMA and physical activity and sleep datasets collected from wearables to assess patients’ quality of life prior to and after a liver transplant?
- Aim 2: What fundamental human factors drive the patients and support person’s acceptance of and sustained engagement with the PeerMA method (as studied by Wulfovich et al [34]).

## Methods

### Overview

This section describes the experimental design of the longitudinal, observational 6-month study. The protocol was designed in close collaboration with a clinical team.

### Study Population

We conducted a longitudinal study of 6 months (autumn of 2019 to spring of 2020) to assess qualitative and quantitative aspects related to the quality of life of adult patients and their caregiving support persons, that is, their family members, neighbors, or friends recruited at the liver transplant clinic of the Stanford University Hospital (Stanford, California). Patients who were in pre- or posttransplant stages would self-report their perceived level of hope [35], quality of sleep [36], fatigue [37], depression [38], and pain [39] using the EMA method. Correspondingly, their support person answered the same questions, leveraging the PeerMA method. Additionally, we collected physical activity and sleep data from those patients (but not from the support persons) using a Fitbit Charge 2 wearable activity monitor.

### Recruitment: Patients and Support Persons

**Figure 1:**
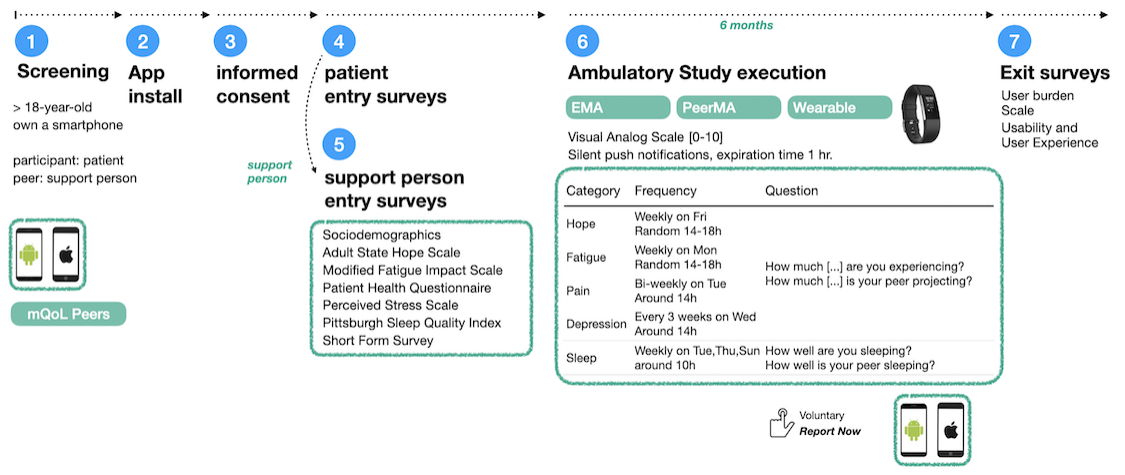
Overview of the study design.

During the screening phase of the study (Figure 1), researchers ensured that participants were at least 18 years old and owned a data-enabled smartphone with a suitable OS version. Participants were patients from the transplant clinic at Stanford University Hospital (Stanford), either in the pre or postliver transplant stage, who were recruited during their scheduled visits to the clinic. The patients usually arrive with their support person, who is one of their informal caregivers (eg, family member, neighbor, or friend). As such, the recruitment of each dyad, consisting of one patient and one support person, was facilitated by the medical team at the hospital, where both patients and support persons were available to be informed about the study and to be enrolled if they consented to participate. Overall, 8 patients participated in this study. Some patients were enrolled before receiving the liver transplant (n=3), while others were enrolled posttransplant (n=5). A total of 7 patients had one support person each, and one patient had no support person. It is a coincidence that all the recruited support persons were spouses of the respective patients. Neither patients nor support persons received monetary compensation in this study. Table 1 shows the number of patients and support persons in the study.

**Table 1:** Socioeconomic characteristics of the study participants (patients and support persons).

Variables			Patients^a^, n (%)		Support persons^a^, n (%)
**Sex**					
	Male	7 (88)		1 (14)	
	Female	1 (13)		6 (86)	
**Age (years)**					
	40-49	0 (0)		1 (14)	
	50-59	1 (13)		1 (14)	
	60-69	5 (63)		2 (29)	
	70-79	0 (0)		0 (0)	
	80 or older	0 (0)		1 (14)	
	Unknown^b^	2 (25)		2 (29)	
**Higher education**					
	High school	1 (13)		1 (14)	
	Bachelor	1 (13)		1 (14)	
	Master	2 (25)		2 (29)	
	Other	2 (25)		1 (14)	
	Unknown^b^	2 (25)		2 (29)	
**Employment status**					
	Employed	1 (13)		2 (29)	
	Retired	5 (63)		3 (43)	
	Unknown^b^	2 (25)		2 (29)	
**Marital status**					
	Married or domestic partnership	8 (100)		7 (100)	

^a^Total number of patients is 8, and the total number of support persons is 7. One patient had no support person.

^b^Did not complete the entry survey.

### Proposed Approach

To explore aim 1, we collected quantitative data such as study participants’ retention during the study, overall agreement between the EMAs and PeerMAs, and qualitative data for the participant’s overall perceived value of the method. The value of the method comes from enabling health care personnel to acquire self-assessments and support person (ie, the observer) assessments paired in time for a more specific assessment of the patient’s health outcomes and quality of care. To explore Aim 2, we gathered qualitative data such as study participants’ reflections after using the method, perceived difficulty in using the technology, and reliability of the data acquisition technologies (smartphones and wearables) that can influence the quality of the collected data.

### Tools

We implemented the PeerMA method by leveraging the mQoL Lab platform [40,41] of the Quality of Life Technologies Lab (University of Geneva). This study used the same toolset from a previous study [3] to collect the EMAs and PeerMAs on the participants’ (patients and support persons) own smartphones. Namely, we developed and published a mobile app called mQoL-Peers that was available for Android and iOS, implementing the EMA and PeerMA methods (further explained in Berrocal et al [41]) with configurable EMA/PeerMA questions’ content and frequency.

### Joining the Study

At the beginning of the study, patients and support persons had a 30-minute face-to-face meeting with a researcher with the following objectives: (1) explain the nature of the study, (2) hand out informed consents, (3) install the mQoL-Peers app and train the participant to use it (concretely, how to complete the entry surveys, and how to address the push notifications for EMA/PeerMA prompts), and (4) answer any questions the participants may have. In particular, we instructed patients to answer the EMA prompts based on how they felt when they completed the EMA. We instructed the support persons to answer the PeerMA prompts based on what they perceived from the patient at the moment when they completed the PeerMA.

### Study Surveys and Self-Reports

This section explains the study entry, ambulatory/continual assessments [42], and study exit surveys. The study entry and exit surveys were administered using Qualtrics (Stanford University), preferred for studies involving clinical patients. The ambulatory EMA and PeerMAs were administered using the mQoL-Peers app.

### Study Entry Surveys

The study entry surveys are summarized in Table 2. Patients and support persons completed the entry surveys before beginning the daily EMA/PeerMA. The study entry survey has two parts: (1) socioeconomic status, including gender, age range, education, marital status, and employment status, and (2) standardized questionnaires that provide a quantitative assessment of health-related outcomes for the patients and support persons.

Regarding the standardized patient-reported outcomes–based questionnaires, we used (1) the 6-item Adult State Hope Scale [35] to measure individual’s agency and pathways to reach goals in life; (2) the 21-item Modified Fatigue Impact Scale [37], a multiple dimension measure of fatigue including physical, cognitive and psychosocial subscales; (3) the 4-item Patient Health Questionnaire [38] which measures depression and anxiety; (4) the 4-item Perceived Stress Scale [43] to measure self-perceived stress of an individual; (5), the 9-item Pittsburgh Sleep Quality Index [36] to measure quality of sleep in clinical populations, and (6) 20-item Short Form Survey [44].

**Table 2:** List of study entry surveys with survey construct, survey name, and reference.

Category	Description
Socioeconomic	Patient’s profile: gender, age range, education, marital and employment status, support person’s profile, and type of relationship with the patient
Hope	6-item Adult State Hope Scale [35]
Fatigue	21-item Modified Fatigue Impact Scale [37]
Depression and anxiety	4-item Patient Health Questionnaire [38]
Stress	4-item Perceived Stress Scale [43]
Sleep quality	12-item Pittsburgh Sleep Quality Index [36]
Medical outcomes	20-item Short Form Survey [44]

### Ambulatory Monitoring: EMA/PeerMA Self-Reporting and Wearable Datasets

The ambulatory surveys that continue as repeated measures in the whole study period are summarized in Table 3. We used single-item questions proposed by Rosenzveig et al [39] on a visual analog scale measuring patients’ levels of sleep, fatigue, hope, pain, and depression since these aspects reflect relevant physical and psychological states of patients during pretransplant disease progression and posttransplant recovery. With EMA, we asked the following questions: “How well are you sleeping?” and “How much {fatigue, hope, pain, depression} are you experiencing?” Correspondingly, with PeerMA, we asked the support person: “How well is your peer sleeping?” and “How much {fatigue, hope, pain, depression} is your peer projecting?” where “the peer” is the patient. For each PeerMA prompt, support persons were able to indicate how confident they were with each assessment on a visual analog scale from 0 to 1, where 0 means “not confident at all,” and 1 means “fully confident.” The selected questions can be studied using introquestive methods [45], and they influence a person’s well-being (eg, high levels of fatigue or bad sleep quality) [46]. Monitoring and early detection of abnormalities in these measured aspects is expected to provide important information to the clinical team as a basis for additional diagnoses or therapeutic decisions.

To issue the EMAs and PeerMAs, we used time-contingent triggers [47] with different application-triggered surveys at different frequencies for each type of question (Table 3). The frequency was chosen by the research team aiming to minimize user burden by implying the collection of several data points during the week instead of, for example, all data points at once. Patients and support persons received the EMA and PeerMA via silent push notifications (no sound and no vibration). Unattended notifications expired after one hour to prevent questions from accumulating, reducing the burden to the patient or support person. However, the smartphone app additionally allowed patients and support persons to initiate EMAs or PeerMAs voluntarily (ie, self-triggered) at any moment they wished to do so.

During the study duration, we also collected physical activity datasets (steps with each minute of data collected categorized as sedentary, light, moderate, or vigorously active) and sleep data (sleep duration) from patients leveraging a Fitbit Charge 2 wearable provided to them.

**Table 3:** List of questions used for the ecological momentary assessments (EMA) and peer-ceived momentary assessments (PeerMA).

Category and questions		Frequency
**Hope**		
	EMA: How much hope are you experiencing? PeerMA: How much hope is your peer projecting?	Weekly on Friday only Random between 2 PM and 6 PM
**Sleep**		
	EMA: How well are you sleeping? PeerMA: How well is your peer sleeping?	Weekly on Tuesday, Thursday, and Sunday Around 10 AM
**Fatigue**		
	EMA: How much fatigue are you experiencing? PeerMA: How much fatigue is your peer projecting?	Weekly on Monday only Random between 2 PM and 6 PM
**Pain**		
	EMA: How much pain are you experiencing? PeerMA: How much pain is your peer projecting?	Bi-weekly on Tuesday only Around 2 PM
**Depression**		
	EMA: How much depression are you experiencing? PeerMA: How much depression is your peer projecting?	Every 3 weeks on Wednesday Around 2 PM

### Exit Survey

At the end of the study, both patients and support persons were invited to complete an exit survey consisting of a subset of 10 questions from the User Burden Scale [48] directly related to the dynamics of this study (level of effort required to use the smartphone app to answer EMAs or PeerMAs). Then, we asked participants to comment on usability aspects of the mobile device app (eg, usability, positive, and negative aspects perceived) and to comment about how patients felt about reflecting on their states during the day. In contrast, support persons answered how they felt about reflecting on their peers’ (ie, the patient) states during the day. Additionally, we asked participants to comment about any other physical or psychological behaviors or states related to health or disease that they would like to assess in the future using these paired self-report-based methods. We also asked patients about their perceived value of the wearable activity monitor during the preparation for the transplant or the recovery period. Finally, we asked their opinion about the study design aspects that could be improved.

### Ethical Considerations

The Panel on Human Subjects in Medical Research of Stanford University approved the study protocol “Studying the Subjective and Objective Momentary Perception of Quality of Life in Different Contexts of Daily Life” (number 47833, Reg# 351). Both participants and support persons signed an informed consent form explaining the implications of their participation in the study. The informed consent contains a description of the overall process, what data they will contribute, and how we will manage such data in time; the time and effort expected from them; associated risks of participating in the study; compensation scheme (there was no compensation in this study); participants’ rights such as withdrawing without any negative consequences; contact information for the participants to approach research personnel other than the front line researchers with whom the participants habitually interact with, among other elements. Additionally, patients signed an authorization form to consent to the use of their health information for research purposes, which is required by the Panel of Human Subjects in Medical Research of Stanford University.

## Results

This section presents the study’s results in 2 subsections after the study aims.

### Aim 1: Feasibility of the Methods in Clinical Settings

This section presents quantitative results related to the feasibility of using the EMA, PeerMA, and physical activity datasets from wearable devices to complement the study of quality-of-life aspects experienced by patients prior to and after receiving an organ transplant. For this feasibility study, we focus on the participation and EMA/PeerMA completion rates, the visual value of the EMA/PeerMA and wearable-based observations, and the similarity scores between the EMA/PeerMA and wearable data streams.

### Collected Data Summary

To begin with, Table 1, extracted from the entry survey, shows the socioeconomic characteristics such as gender, age, marital status, education, and employment. Table 4 summarizes the questionnaire/test scores derived for patients and support persons at the beginning of the study, before the collection of daily EMAs and PeerMAs started.

**Table 4:** Patients’ and support persons’ scores computed from the study entry surveys (no data available for P4 and SP4).

Instrument/survey name and subscales		Respondents (Px=Patient x, SPx=Support Person x)											
		P1	SP1	P2	SP2	P3	SP3	P5	SP5	P6	SP6	P7	SP7
**Adult State Hope Scale [35]^a^**													
	Pathways	24	23	21	24	23	22	21	N/A^b^	23	22	22	17
	Agency	24	21	22	22	21	22	16	N/A	22	23	19	18
	Total	48	44	43	46	44	44	37	N/A	45	45	41	35
**Modified Fatigue Impact Scale [37]^c^**													
	Physical	16	14	22	3	35	1	5	N/A	27	3	22	N/A
	Social	19	11	4	3	25	2	8	N/A	4	1	19	N/A
	Psychosocial	0	1	4	1	8	0	0	N/A	5	2	4	N/A
	Total	35	26	30	7	68	3	13	N/A	36	6	45	N/A
**Patient Health Questionnaire [38]^d^**													
	Anxiety	1	0	1	0	1	0	1	N/A	1	2	0	1
	Depression	1	0	1	0	2	0	0	N/A	2	0	0	2
	Total	2	0	2	0	3	0	1	N/A	3	2	0	3
**Perceived Stress Scale [43]^e^**													
	Total	2	0	5	2	7	2	3	N/A	4	4	3	7
**Pittsburgh Sleep Quality Index [36]^f^**													
	Subjective sleep quality	0	0	1	0	3	1	1	N/A	1	0	1	N/A
	Sleep latency	1	0	3	1	2	1	0	N/A	1	0	0	N/A
	Sleep duration	1	0	2	1	1	0	1	N/A	1	0	1	N/A
	Habitual sleep efficiency	1	1	3	0	1	1	1	N/A	1	1	0	N/A
	Sleep disturbances	1	1	1	1	1	1	1	N/A	1	1	2	N/A
	Use of sleep medication	0	0	0	0	0	0	0	N/A	1	0	0	N/A
	Daytime dysfunction	1	0	0	0	3	0	0	N/A	1	0	3	N/A
**Short Form Survey [44]^g^**													
	Physical functioning	83	100	25	100	33	100	83	N/A	33	100	58	67
	Role functioning	0	100	75	100	0	100	100	N/A	0	100	100	50
	Social functioning	100	100	20	100	0	100	100	N/A	0	100	80	80
	Mental health	88	92	72	84	76	88	72	N/A	60	64	92	56
	Health perceptions	72	77	17	95	22	100	62	N/A	42	92	42	25
	Pain	20	40	60	0	80	20	20	N/A	60	20	60	80

^a^In this scale, higher scores indicate higher hope levels.

^b^Not applicable.

^c^In this scale, higher scores indicate a greater impact of fatigue on a persons’ activities.

^d^In this scale, scores are rated as normal (0-2), mild (3-5), moderate (6-8), and severe (9-12).

^e^In this scale, higher scores are correlated with more stress.

^f^In this scale, scoring is based on a 0 to 3 scale, whereby 3 reflects the negative extreme on the Likert Scale.

^g^In this scale, 0 is the lowest score and 100 is the highest. Higher scores indicate better health except for pain.

In this study, we collected 2118 participant-days, 1142 patient-days (mean 143, SD 63), and 976 support person–days (mean 139, SD 77). Table 5 shows the days each patient and support person contributed to the study. The value of column “Expected number of EMA/PeerMA” is calculated based on the number of weeks the person was in the study and the number of surveys that should have been triggered by that time. Patients received 821 EMA prompts (mean 103, SD 53) and responded a total of 514 EMAs (mean 64, SD 50) with an average response rate of 63% (SD 26%). Support persons received 612 PeerMA prompts (mean 87, SD 60) and responded to a total of 448 PeerMAs (mean 64, SD 52) with an average response rate of 74% (SD 39%). The number of answered EMA/PeerMAs is divided into application-triggered (prompts presented by the app at the programmed frequency for each question) and self-triggered (those initiated by patients and support persons voluntarily at any moment). Table 5 also shows the response rate, computed as a percentage of the answered EMAs/PeerMAs over the expected number of EMAs/PeerMAs. As Table 5 shows, the response rate can be above 100% when the patients or support persons answer almost all application-triggered surveys, and additionally, they answer many self-triggered surveys, sometimes making up for missed application-triggered surveys and sometimes contributing voluntarily with an additional data point.

**Table 5:** Summary of the data contributed by each patient and support person in the study.

Patient/ support person ID	Time in the study, days (weeks)	Expected number of EMA^a^/PeerMA^b^	EMA/PeerMA answered			Response rate (%)		Data from wearable	
			Application triggered	Self-triggered			Physical activity (days)		Sleep data (days)
Patient 1	196 (28.0)	164	161	0	98		196		191
Support person 1	196 (28.0)	164	147	0	90		N/A^c^		N/A
Patient 2	169 (24.1)	140	0	69	49		127		95
Support person 2	169 (24.1)	140	0	91	65		N/A		N/A
Patient 3	39 (5.6)	30	1	29	100		39		15
Support person 3	39 (5.6)	30	4	13	57		N/A		N/A
Patient 4	76 (10.9)	59	2	26	47		11		4
Support person 4	76 (10.9)	59	9	61	119		N/A		N/A
Patient 5	95 (13.6)	77	32	1	43		47		18
Support person 5	94 (13.4)	77	27	71	127		N/A		N/A
Patient 6	174 (24.9)	140	17	25	30		0		0
Support person 6	162 (23.1)	135	14	7	16		N/A		N/A
Patient 7	69 (9.9)	53	0	32	60		0		0
Support person 7	12 (1.7)	7	0	4	57		N/A		N/A
Patient 8	195 (27.9)	158	117	2	75		2		2
Support person 8	N/A	N/A	N/A	N/A	N/A		N/A		N/A

^a^EMA: ecological momentary assessment.

^b^PeerMA: peer-ceived momentary assessment.

^c^Not applicable.

As noted in Table 5, not all patients and support persons remained in the study for the expected period of 6 months (approximately 26 weeks). During the exit interview, some of them stated various reasons why they had stopped contributing. For instance, some patients said they felt very well and healthy and even ahead of the recovery process, and thus, they did not perceive much value from answering the EMA/PeerMA prompts. Additionally, some support persons indicated that answering the questions during working hours was challenging, and others indicated that they did not see EMA/PeerMA notifications at all for some time, which could be due to some changes in their smartphone settings.

For each patient and support person, we normalized their EMA/PeerMA responses to 0 to 1 based on the highest and lowest assessment given by each person. Table 6 shows the median, mean, and SD of all assessments for hope, sleep quality, fatigue, depression, and pain. In Table 6, lower values in the “Median” and “Mean” columns represent more desirable states in the case of fatigue, depression, and pain, and less desirable states in the case of hope and sleep quality. Conversely, higher values in the “Median” and “Mean” represent less desirable states for fatigue, depression, and pain, and more desirable states for hope and sleep quality.

Table 7 compares the concordance between the patient’s and the support person’s reports. It shows the number of dyads for which the absolute difference between the mean of each quality of life aspect was under a low threshold of 15% proposed as a minimal importance difference by the Institute for Quality and Efficiency in Health Care in Germany [49].

**Table 6:** Summary of the ecological momentary assessment (EMA) and peer-ceived momentary assessment (PeerMA) aggregated values obtained from patients and support persons. Each row shows the median, mean, and SD for the corresponding patient or support person calculated from all the assessments issued by that person.

Patient / support person ID	Hope (0-1) (higher is better)			Sleep quality (0-1) (higher is better)			Fatigue (0-1) (lower is better)			Depression (0-1) (lower is better)			Pain (0-1) (lower is better)	
	Median	Mean (SD)	Median		Mean (SD)	Median		Mean (SD)	Median		Mean (SD)	Median		Mean (SD)
Patient 1	0.69	0.64 (0.3)	0.89		0.82 (0.17)	0.08		0.23 (0.29)	0.30		0.31 (0.32)	0.25		0.34 (0.29)
Support person 1	0.81	0.71 (0.29)	0.75		0.72 (0.14)	0.35		0.35 (0.25)	0.14		0.31 (0.38)	0.10		0.21 (0.28)
Patient 2	0.69	0.6 (0.31)	0.85		0.72 (0.28)	0.35		0.43 (0.35)	0.34		0.4 (0.3)	0.27		0.38 (0.34)
Support person 2	0.81	0.64 (0.3)	0.91		0.76 (0.29)	0.12		0.27 (0.26)	0.10		0.28 (0.28)	0.21		0.32 (0.27)
Patient 3	0.69	0.64 (0.32)	0.34		0.44 (0.32)	0.19		0.29 (0.26)	0.18		0.31 (0.28)	0.19		0.3 (0.28)
Support person 3	0.69	0.64 (0.32)	0.46		0.5 (0.37)	0.14		0.22 (0.27)	0.21		0.29 (0.3)	0.16		0.21 (0.28)
Patient 4	0.61	0.59 (0.24)	0.76		0.69 (0.29)	0.16		0.35 (0.36)	0.12		0.19 (0.21)	0.19		0.31 (0.32)
Support person 4	0.75	0.64 (0.24)	0.78		0.69 (0.28)	0.52		0.55 (0.21)	0.51		0.53 (0.26)	0.61		0.59 (0.23)
Patient 5	0.50	0.5 (0.5)	0.88		0.77 (0.29)	0.86		0.62 (0.54)	0.50		0.5 (0.71)	0.00		0.25 (0.5)
Support person 5	0.69	0.66 (0.14)	0.77		0.75 (0.15)	1.00		0.94 (0.2)	1.00		0.87 (0.27)	0.00		0.12 (0.27)
Patient 6	0.41	0.47 (0.31)	0.66		0.6 (0.26)	0.75		0.6 (0.29)	0.44		0.36 (0.24)	0.36		0.39 (0.3)
Support person 6	0.71	0.63 (0.34)	0.43		0.52 (0.37)	0.73		0.61 (0.4)	0.07		0.27 (0.36)	0.74		0.57 (0.41)
Patient 7	0.81	0.77 (0.24)	0.75		0.72 (0.18)	0.70		0.63 (0.29)	0.34		0.33 (0.2)	0.59		0.63 (0.28)
Support person 7	0.29	0.39 (0.49)	0.86		0.68 (0.46)	0.45		0.48 (0.45)	0.36		0.43 (0.49)	0.27		0.39 (0.47)

**Table 7:** Distribution of the number of dyads with an absolute difference of up to 15% between ecological momentary assessment (EMA) and peer-ceived momentary assessment (PeerMA).

Absolute difference between EMA/PeerMA Means	Number of dyads (from total of 7 dyads)				
	Hope, n (%)	Sleep, n (%)	Fatigue, n (%)	Depression, n (%)	Pain, n (%)
≤5%	3 (43)	4 (57)	1 (14)	2 (29)	0 (0)
≤10%	1 (14)	3 (43)	1 (14)	2 (29)	2 (29)
≤15%	0 (0)	0 (0)	2 (29)	1 (14)	2 (29)
>15%	3 (43)	0 (0)	3 (43)	2 (29)	3 (43)

### Visual Exploration of Daily EMA/PeerMA Dynamics

#### Overview

This section shows selected visualizations of the values reported by patients and support persons via EMA/PeerMA surveys, as well as physical activity datasets collected from a wearable activity monitor by the patient. The intention of the plots is to aid in understanding the magnitude of agreement/disagreement in their EMA/PeerMA self-reported assessments over time.

To use the collected EMA/PeerMAs and wearable-based physical activity data, we performed a few data preparation steps. First, we searched for duplicate EMA/PeerMAs assessments (issued on the same day) and removed incomplete EMA/PeerMAs—if at least one of them was complete. If both duplicate assessments were complete or incomplete, we removed the oldest one (they usually differed only by a few seconds/minutes). Second, we aligned the patient and support person EMA/PeerMAs data streams to a common start and end date. To do this, we used the first day of the patient’s data stream as the start (trimming the support person’s data stream to match the patient’s start date), and for the end date, we picked the day from either data stream that resulted in the largest common set of assessments from both participants. In other words, we used the earliest date among the last patient report and the last support person’s report. Third, we imputed missing EMA/PeerMA values using a linear regression model with polynomial features of second degree and the Ridge algorithm with default parameters in the Scikit-learn data analytics framework. We selected this model based on prior work [50] and the fact that it produces smooth curves with suitable values according to our datasets.

#### All Outcomes: Visual Comparison

**Figure 2:**
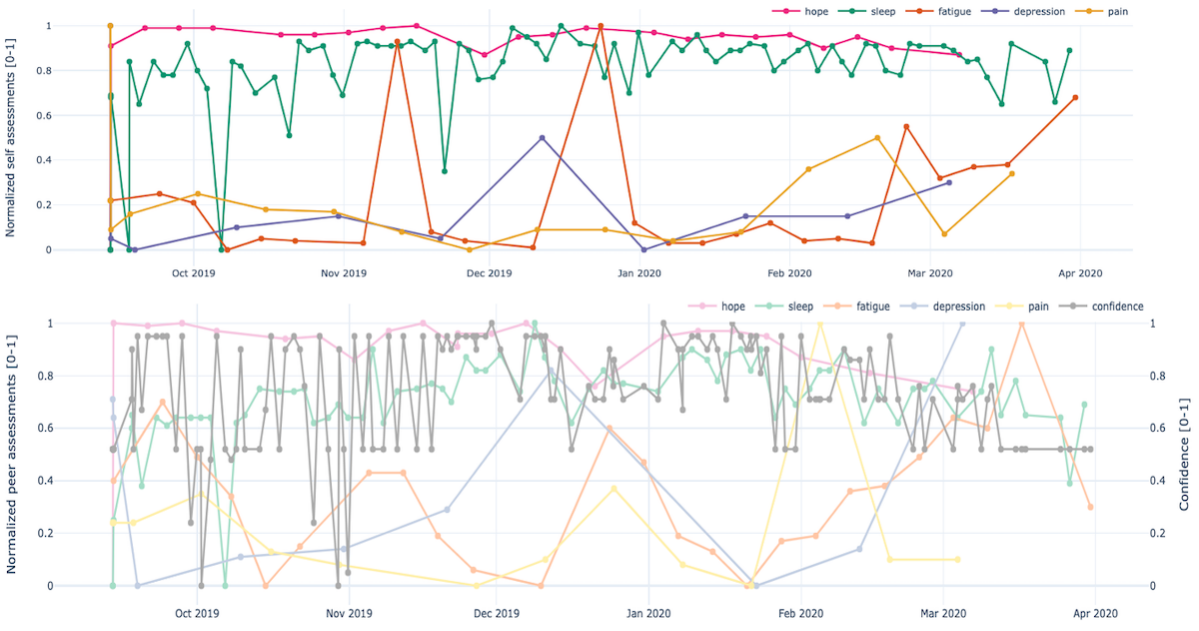
Sample plots for the combined ecological momentary assessment (EMA)/ peer-ceived momentary assessments (PeerMAs) for patient 1-support person 1. The x-axis represents the time in the study, and the y-axis represents the magnitude of the normalized assessments for hope, sleep quality, fatigue, depression, and pain. The upper plot corresponds to the patient, and the lower plot corresponds to the support person.

Figure 2 shows a sample chart for the dyad (patient 1, support person 1).

**Figure 3:**
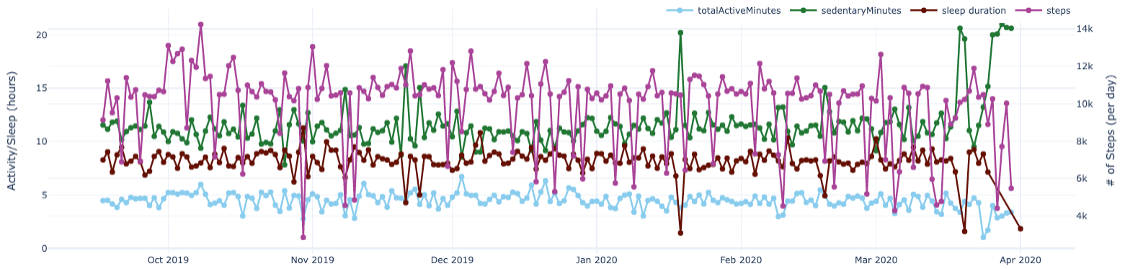
Sample plot for patient 1’s physical activity data. The x-axis represents the time in the study, and the y-axis represents the magnitude of the absolute values collected from the wearable. The primary y-axis (left) represents hours for measures such as sleep duration and active time of the patient. The secondary y-axis (right) represents the absolute number of steps measured from the patient.

Figure 3 shows the plot of physical activity data for patient 1 collected by the wearable activity monitor for the same period of time.

#### Outcome-by-Outcome Comparison

**Figure 4:**
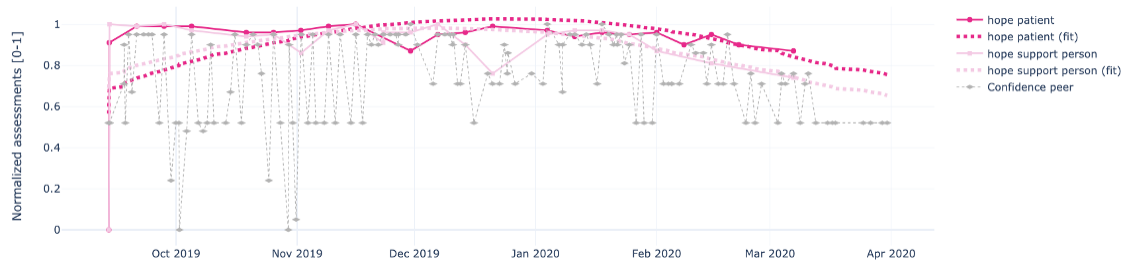
Sample plots for individual ecological momentary assessment (EMA)/ peer-ceived momentary assessments (PeerMAs) related to hope. The x-axis represents the time in the study, and the y-axis represents the magnitude of the normalized assessments. The solid spheres represent the acquired values, and the dotted squares represent the regression curves. The light gray diamonds represent the confidence value estimated by the support person each time they issued a PeerMA.

**Figure 5:**
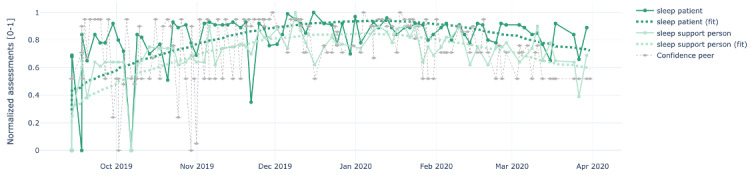
Sample plots for individual ecological momentary assessment (EMA)/ peer-ceived momentary assessments (PeerMAs) related to sleep quality. The x-axis represents the time in the study, and the y-axis represents the magnitude of the normalized assessments. The solid spheres represent the acquired values, and the dotted squares represent the regression lines. The light gray diamonds represent the confidence value estimated by the support person each time they issued a PeerMA.

**Figure 6:**
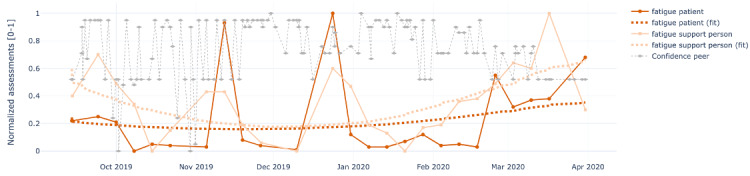
Sample plots for individual ecological momentary assessment (EMA)/ peer-ceived momentary assessments (PeerMAs) related to fatigue. The x-axis represents the time in the study, and the y-axis represents the magnitude of the normalized assessments. The solid spheres represent the acquired values, and the dotted squares represent the regression lines. The light gray diamonds represent the confidence value estimated by the support person each time they issued a PeerMA.

**Figure 7:**
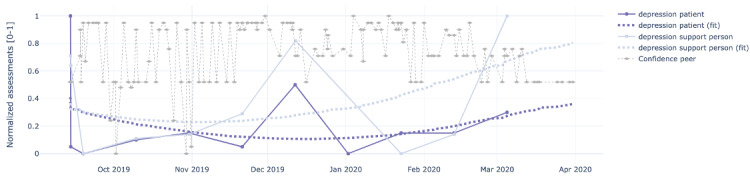
Sample plots for individual ecological momentary assessment (EMA)/ peer-ceived momentary assessments (PeerMAs) related to depression. The x-axis represents the time in the study, and the y-axis represents the magnitude of the normalized assessments. The solid spheres represent the acquired values, and the dotted squares represent the regression lines. The light gray diamonds represent the confidence value estimated by the support person each time they issued a PeerMA.

**Figure 8:**
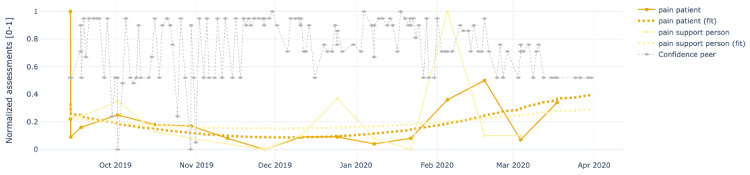
Sample plots for individual ecological momentary assessment (EMA)/ peer-ceived momentary assessments (PeerMAs) related to pain. The x-axis represents the time in the study, and the y-axis represents the magnitude of the normalized assessments. The solid spheres represent the acquired values, and the dotted squares represent the regression lines. The light gray diamonds represent the confidence value estimated by the support person each time they issued a PeerMA.

Figures 4-8 show the individual charts for the dyad (patient 1, support person 1) corresponding to each of the constructs separately for hope, sleep quality, fatigue, depression, and pain. Stronger colors represent the patient’s assessments, while lighter colors represent the support person’s assessments. The light gray line represents the confidence value estimated by the support person each time they issued a PeerMA.

In Figure 4, we can see a high similarity regarding the perception of hope between the patient and the support person along the timeline. In Figure 5, the patient reported a low value for sleep quality on October 6 and November 21; when we looked at the patient’s comments for those days, we learned that one was due to insomnia, and the second one was due to anxiety caused by a meeting the next day. Likewise, in Figure 6, the patient reported a high value for fatigue on November 11 and December 23. High fatigue for a recovering patient could be a sign of alert at times. In this case, the patient’s comments reflect that fatigue is coming from usual daily life activities and social activities like entertaining house guests.

Figure 7 shows that on December 11, both the patient and support person reported a value higher than usual for depression. In this case, the patient’s comment is, however, rather positive, indicating that everything was going well, hence not aligned with the self-reported level of depression. This might also be a sign of alert for caregivers; however, as the plot shows, subsequent reports by the patient return to lower values, suggesting that perhaps it was an isolated event that triggered a higher perception of depression. Regarding the perception of pain shown in Figure 8, there are mainly 2 moments when the patient and support person scores differ more: December 23 and February 4. Although there are no comments for these reports, from other patients’ comments, we learned that the patient has comorbidities that may affect the perception of physical pain.

#### Outcome-by-Outcome and Wearable Outcomes Comparison

**Figure 9:**
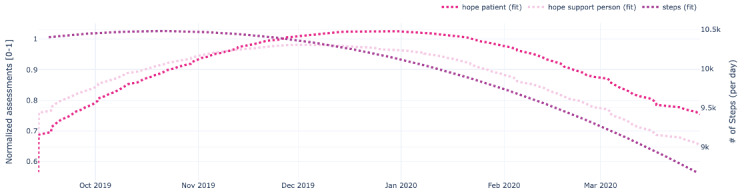
Sample plots for physical activity and individual ecological momentary assessment (EMA)/ peer-ceived momentary assessments (PeerMAs) related to hope. The x-axis represents the time in the study. The primary y-axis (left) represents regressed curves (pink) for hope by the patient and support person. The secondary y-axis (right) represents the regressed curve (purple) for the number of steps measured by the patient.

**Figure 10:**
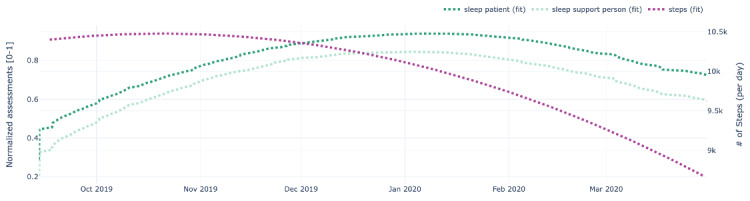
Sample plots for physical activity and individual ecological momentary assessment (EMA)/ peer-ceived momentary assessments (PeerMAs) related to sleep quality. The x-axis represents the time in the study. The primary y-axis (left) represents regressed curves (green) for sleep quality by the patient and support person. The secondary y-axis (right) represents the regressed curve (purple) for the number of steps measured by the patient.

**Figure 11:**
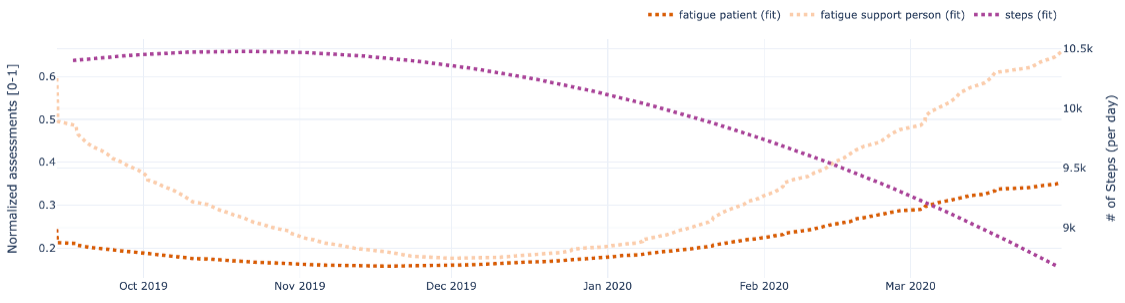
Sample plots for physical activity and individual ecological momentary assessment (EMA)/ peer-ceived momentary assessments (PeerMAs) related to fatigue. The x-axis represents the time in the study. The primary y-axis (left) represents regressed curves (orange) for fatigue by the patient and support person. The secondary y-axis (right) represents the regressed curve (purple) for the number of steps measured by the patient.

**Figure 12:**
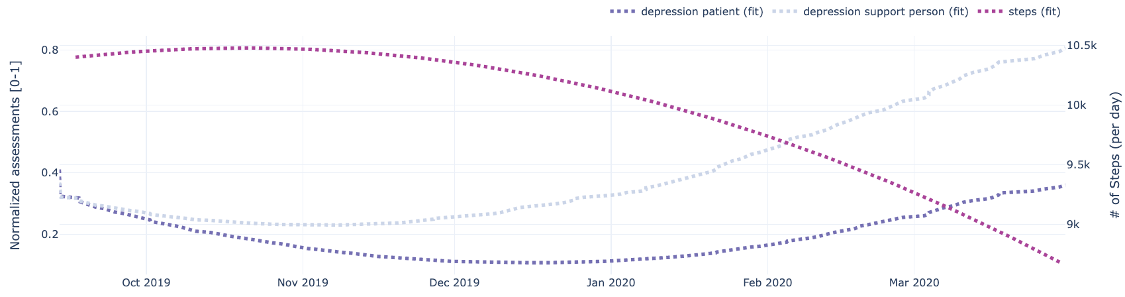
Sample plots for physical activity and individual ecological momentary assessment (EMA)/ peer-ceived momentary assessments (PeerMAs) related to depression. The x-axis represents the time in the study. The primary y-axis (left) represents regressed curves (blue) for depression by the patient and support person. The secondary y-axis (right) represents the regressed curve (purple) for the number of steps measured by the patient.

**Figure 13:**
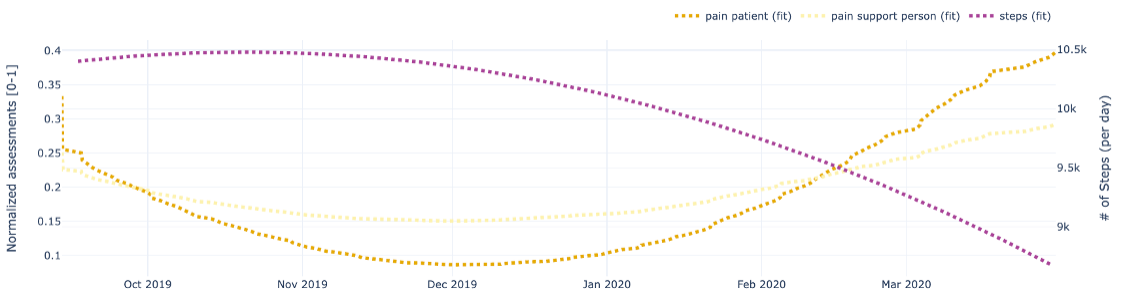
Sample plots for physical activity and individual ecological momentary assessment (EMA)/ peer-ceived momentary assessments (PeerMAs) related to pain. The x-axis represents the time in the study. The primary y-axis (left) represents regressed curves (yellow) for pain by the patient and support person. The secondary y-axis (right) represents the regressed curve (purple) for the number of steps measured by the patient.

Figures 9-13 also show data for the dyad (patient 1, support person 1), focusing on the regressed curves for the EMAs and PeerMAs (hope, sleep quality, fatigue, depression, and pain) along one physical activity marker collected from the patient corresponding to the number of steps walked per day. Table 8 shows the actual correlations between EMA scores and the patient’s physical activity, which in this case are moderate to high, both positive and negative.

**Table 8:** Spearman correlations between ecological momentary assessment (EMA) and physical activity (number of steps) collected for each patient in the study. Each row shows the correlation between the patients’ self-assessments and the number of steps recorded with the wearable activity monitor, and each column represents a construct of the study.

Dyad	Hope		Sleep			Fatigue			Depression			Pain	
	rs	*P* value	rs	*P* value	rs		*P* value	rs		*P* value	rs		*P* value
1	0.96	<.001	–0.49	<.001	–0.61		<.001	0.98		<.001	–0.56		.04
2	0.97	<.001	0.79	<.001	–0.99		<.001	–0.98		<.001	–0.95		<.001
3	0.18	.40	0.29	.17	–0.80		<.001	0.39		.06	–0.48		.02
4	0.01	.94	1.00	<.001	–0.72		<.001	–0.95		<.001	–0.77		<.001
5	0.91	<.001	0.76	<.001	–0.85		<.001	–0.83		<.001	–0.88		<.001

### Similarity Scores

#### Overview

To assess the value of the scores given by the patient and the support person, we used 2 approaches: similarity of the EMA and PeerMA scores in time, using standard distance-based metrics, and similarity scores based on statistical correlation analysis.

#### PeerMA as a Model of EMA

We wanted to measure how close or similar the regressed curves between the patient and the support person were for all the health outcomes they assessed during the study. To do this, we took all the data pairs for each dyad (patient, support person) and computed the following similarity metrics: mean absolute error (MAE), mean squared error, root-mean-squared error, and *R*^2^. These metrics are commonly used to assess the fitness of a regression model, in other words, to assess how well a regression model represents the data points from which it was sourced. However, in our case, because we have regression curves representing both the patient and the support person’s data points, we consider the curve created with the patients’ assessments to be the ground truth and the curve created with the support person’s assessments as the approximation model.

Table 9 shows the value for all the similarity scores for each dyad (Dyad X represents the pair [PatientX, Support PersonX]). For example, if we focus on the first segment of Table 9, “Hope,” we see that the error (MAE) or mean difference between patient 1’s assessment of hope and that of his/her peer, support person 1, was about 4% (0.0412). This value is located under the “Dyad 1” column and can be visually inspected in Figure 4. For dyad 2, that difference was slightly smaller, 3.5% (0.0350), meaning that both patients and support persons in dyads 1 and 2 reported similar values in their assessments. On the contrary, dyad 7 shows a difference of nearly 74% (0.7377), meaning that the curves representing patient 7’s and support person 7’s assessments of hope are very dissimilar.

**Table 9:** Summary of ecological momentary assessment (EMA)/peer-ceived momentary assessment (PeerMA) similarity scores: mean absolute error (MAE), mean squared error (MSE), root-mean-squared error (RMSE), and R2.

Aspect and metric			Dyad 1		Dyad 2		Dyad 3		Dyad 4		Dyad 5	Dyad 6		Dyad 7
**Hope**														
	MAE	0.0412		0.0350		0.1589		0.1487		0.2583		0.2970	0.7377	
	MSE	0.0022		0.0016		0.0542		0.0317		0.0829		0.1185	0.5661	
	RMSE	0.0469		0.0400		0.2329		0.1780		0.2878		0.3442	0.7524	
	*R^2^*	0.9346		0.9807		–1.8358		–2.1791		–1.3084		–1.6667	–39.5400	
**Sleep**														
	MAE	0.0979		0.0565		0.2474		0.0315		0.1030		0.2321	0.0507	
	MSE	0.0100		0.0046		0.0988		0.0014		0.0130		0.0740	0.0039	
	RMSE	0.0998		0.0676		0.3143		0.0371		0.1141		0.2720	0.0628	
	*R^2^*	–0.6469		0.9288		–15.3476		0.9685		–0.6926		–13.9986	–2.7936	
**Fatigue**														
	MAE	0.1202		0.0919		0.0989		0.2211		0.5018		0.2356	0.0992	
	MSE	0.0199		0.0188		0.0128		0.0601		0.3857		0.0701	0.0163	
	RMSE	0.1410		0.1369		0.1129		0.2452		0.6210		0.2648	0.1279	
	*R^2^*	–1.7331		0.8311		–0.3196		–2.0013		–1.1524		–1.2381	–0.1915	
**Depression**														
	MAE	0.1475		0.0611		0.1198		0.3004		0.6210		0.2508	0.3307	
	MSE	0.0280		0.0050		0.0200		0.1011		0.4578		0.0810	0.1187	
	RMSE	0.1672		0.0704		0.1415		0.3179		0.6766		0.2847	0.3445	
	*R^2^*	–1.2353		0.9433		–5.6800		–4.1741		–2.1509		–776.5628	–14.0340	
**Pain**														
	MAE	0.1443		0.0758		0.1295		0.2076		0.3401		0.1951	0.5581	
	MSE	0.0367		0.0096		0.0202		0.0510		0.2138		0.0489	0.3352	
	RMSE	0.1915		0.0978		0.1422		0.2258		0.4624		0.2213	0.5790	
	*R^2^*	–0.6389		0.9077		–13.4371		–261.7243		–0.3047		–3.8061	–18.1295	

#### EMA/PeerMA Spearman Correlations

We performed a correlation analysis for EMA/PeerMA values for the various states without assuming that EMA and PeerMA measure the same constructs. We applied the Spearman rank correlation method because (1) patient and support person assessments are not independent; they both refer to the state of the patient, and (2) the Shapiro-Wilk, D’Agostino K2, and Anderson-Darling tests indicate that not all patients’ and support persons’ assessments were normally distributed.

The Spearman rank correlation coefficients for each of the states are presented in Table 10. Each row presents the correlation coefficient rs and *P* value for a patient-support person dyad. For example, the correlation between patient 1’s assessment of hope and that of his/her peer, support person 1, is 0.96 (highly positive). Table 11 presents a summary of the values from Table 10, classified into 6 correlation groups. The correlations are predominantly on the positive side; 77% of the correlations are positive (46% high, 20% moderate, and 11% weak), and 23% of the correlations are negative (9% high, 3% moderate, and 11% weak).

**Table 10:** Spearman correlations between ecological momentary assessment (EMA) and peer-ceived momentary assessment (PeerMA) values collected for each study dyad. Each row shows the correlation between the patients’ EMAs and support persons’ PeerMAs, and each column represents a construct of the study.

Dyad	Hope		Sleep		Fatigue		Depression		Pain	
	rs	*P* value	rs	*P* value	rs	*P* value	rs	*P* value	rs	*P* value
1	0.96	<.001	0.97	<.001	0.88	<.001	0.78	.01	0.90	<.001
2	0.98	<.001	0.81	<.001	1.00	<.001	1.00	<.001	0.96	<.001
3	0.18	.40	0.29	.17	0.80	<.001	–0.13	.56	0.48	.02
4	0.11	.50	0.92	<.001	0.71	<.001	0.78	<.001	–0.23	.16
5	0.90	<.001	–0.71	<.001	–1.00	<.001	0.80	<.001	–0.81	<.001
6	0.62	<.001	0.15	.52	–0.26	.22	0.54	.01	0.22	.36
7	–0.50	.01	0.07	.72	0.05	.79	–0.23	.25	0.52	.01

**Table 11:** Summary of ecological momentary assessment (EMA)/ peer-ceived momentary assessment (PeerMA) Spearman correlations from Table 10.

Correlation strength	Hope, n (%)	Sleep, n (%)	Fatigue, n (%)	Depression, n (%)	Pain, n (%)	Total, n (%)
Highly positive^a^	3 (43)	3 (43)	4 (57)	4 (57)	2 (29)	16 (46)
Moderately positive^b^	1 (14)	3 (43)	0 (0)	1 (14)	2 (29)	7 (20)
Weakly positive^c^	2 (29)	0 (0)	1 (14)	0 (0)	1 (14)	4 (11)
Weakly negative^d^	0 (0)	0 (0)	1 (14)	2 (29)	1 (14)	4 (11)
Moderately negative^e^	1 (14)	0 (0)	0 (0)	0 (0)	0 (0)	1 (3)
Highly negative^f^	0 (0)	1 (14)	1 (14)	0 (0)	1 (14)	3 (9)

^a^(0.67 to 1.00): Values of the Spearman correlation inside this interval are considered highly positive.

^b^(0.34 to 0.66): Values of the Spearman correlation inside this interval are considered moderately positive.

^c^(0.00 to 0.33): Values of the Spearman correlation inside this interval are considered weakly positive.

^d^(–0.33 to 0.00): Values of the Spearman correlation inside this interval are considered weakly negative.

^e^(– 0.66 to –0.34): Values of the Spearman correlation inside this interval are considered moderately negative.

^f^(–1.00 to –0.67): Values of the Spearman correlation inside this interval are considered highly negative.

#### EMA/Physical Activity Spearman Correlations

We performed a correlation analysis between the EMA values for the various states and physical activity (number of steps) measured by a wearable activity monitor. We applied the Spearman rank correlation method because (1) patient and support person assessments are not independent; they both refer to the state of the patient, and (2) the Shapiro-Wilk, D’Agostino K2, and Anderson-Darling tests indicate that not all patients’ and support persons’ assessments were normally distributed.

The Spearman rank correlation coefficients for each of the states are presented in Table 8, excluding dyads 6 and 7 because we did not collect physical activity records for those patients. Each row presents the correlation coefficient rs and *P* value for a patient-support person dyad. Table 12 presents a summary of the values from Table 8, classified into 6 correlation groups. In this case, the correlations are balanced between positive and negative. We observe that 44% of the correlations are positive (28% high, 4% moderate, and 12% weak), while 56% of the correlations are negative (40% high, 16% moderate, and 0% weak).

**Table 12:** Summary of ecological momentary assessment (EMA)/steps Spearman correlations from Table 8.

Correlation strength	Hope, n (%)	Sleep, n (%)	Fatigue, n (%)	Depression, n (%)	Pain, n (%)	Total, n (%)
Highly positive^a^	3 (60)	3 (60)	0 (0)	1 (20)	0 (0)	7 (28)
Moderately positive^b^	0 (0)	0 (0)	0 (0)	1 (20)	0 (0)	1 (4)
Weakly positive^c^	2 (40)	1 (20)	0 (0)	0 (0)	0 (0)	3 (12)
Weakly negative^d^	0 (0)	0 (0)	0 (0)	0 (0)	0 (0)	0 (0)
Moderately negative^e^	0 (0)	1 (20)	1 (20)	0 (0)	2 (40)	4 (16)
Highly negative^f^	0 (0)	0 (0)	4 (80)	3 (60)	3 (60)	10 (40)

^a^(0.67 to 1.00): Values of the Spearman correlation inside this interval are considered highly positive.

^b^(0.34 to 0.66): Values of the Spearman correlation inside this interval are considered moderately positive.

^c^(0.00 to 0.33): Values of the Spearman correlation inside this interval are considered weakly positive.

^d^(–0.33 to 0.00): Values of the Spearman correlation inside this interval are considered weakly negative.

^e^(– 0.66 to –0.34): Values of the Spearman correlation inside this interval are considered moderately negative.

^f^(–1.00 to –0.67): Values of the Spearman correlation inside this interval are considered highly negative.

### Aim 2: Human Factors

This section presents human factors driving the acceptance of and the sustained engagement with the methods (as studied by Wulfovich et al [34]). Because the PeerMA method is inspired by 2 related concepts, observers or proxies from clinical research [13], and the existing EMA method [1], it is expected that some of the human factors driving the acceptance of PeerMA are similar to or related to the human factors driving the acceptance of EMA. We report qualitative data such as participants’ reflections after using EMA and PeerMA, the perceived difficulty of using the technology (smartphone app), and the reliability of the technologies to preserve the quality of the collected data. Results were drawn from patients’ and support persons’ comments typed in when they completed an EMA/PeerMA and via the exit survey at the end of the study. Below, we list the relevant human factors identified in this study that impact the acceptance of the PeerMA method.

### Self-Awareness and the Value Proposition of the EMA/PeerMA Methods

One important factor determining participants’ engagement in the study and motivation to contribute EMA/PeerMA datasets was their prior knowledge and self-awareness about the health and disease-related states we studied (hope, sleep, fatigue, depression, and pain). Patients who are not experiencing negative moods (low hope and depression), or poor physical states (bad sleep, fatigue, or pain) may be less likely to contribute inputs with the EMA or PeerMA methods as valuable sources for better clinical decision-making. These patients may have already communicated their positive state to the clinical team and do not see a value in communicating more. So, the perceived added value of using such a method is a factor that influences participants’ acceptance. In general, most patients in the study expressed positive attitudes towards their health during their recovery stage. They exhibited a positive mindset and were committed to following medical indications such as walking and procuring healthy sleep habits. As a result, after a few weeks in the recovery process, many of them indicated that the mobile app was not providing them value. They may have assumed that their current clinical decisions are accurate, as they result in their rather positive state, hence no EMA or PeerMA data is necessary.

The second important factor is to provide feedback or brief information to the participants. In this study, the patients were informed that the EMA or PeerMA served for data collection only, and the mobile app did not display any additional information to the participants or to the clinicians. That was a methodological decision in this preliminary study because one needs to carefully design what and how to show patients and peers this sensitive information about their health. However, that decision reduced the motivation to continue completing EMA and PeerMA for some participants. Unlike our app, the Fitbit app companion that some participants used allowed them to monitor their physical activity and sleep goals, something none of them had experienced before. In fact, all patients who use Fitbit wearables have expressed positive comments about it. The comments from patients included expressions such as “Feel great, loving the push I’m getting from using the Fitbit,” “I liked the Fitbit, very informative on my progress,” and “It is helpful to know how much exercise I get, and to know about my heart rate during various times of the day.”

### Individual’s Ability, Motivation, and Availability for PeerMA

The acceptance of the PeerMA method is also influenced by how confident individuals feel in using it, based on their smartphone use skills or abilities to perform the assessment task via the EMA/PeerMA method. Another factor is related to time constraints and availability during the day to attend the assessment task via the EMA/PeerMA methods. This is particularly important for patients who may be away from their smartphones during certain hours due to clinical visits, rehabilitation, and rest, among other duties and daily life activities. In this study, we did not impose rules upon the methods or strategies support persons would use to complete their assessments. Unlike patients, the support persons did not express any challenges related to performing the task of providing assessments about someone else.

Because EMA and PeerMA-based studies require that participants invest a bit of time to complete the assessments, it is critical that the instruments involved (eg, smartphone app and wearable devices) are simple and reliable to operate. In our case, participants expressed satisfaction because our study had low complexity, for example, “It was easy to use… though didn’t always see it till later” (patient), “It was a positive experience to share” (support person). Nevertheless, they also criticized the fact that the mobile app becomes boring to use after a few days “the only thing I did not like was every day it was the same questions. Needs more variety” (patient), “Add some variety to the questions each time, more dynamic or based on previous responses such as: yesterday you reported feeling down, how are you feeling today, better, same worse?” (patient).

### Usability and Potential Participant Burden of the EMA/PeerMA Methods

Even though the focus of this study was on the PeerMA method and not on the strengths and features of the mQoL-Peers mobile app operationalizing the EMA/PeerMA, participants reported usability aspects of the app. The mQoL-Peers smartphone apps were easy to use, as attested by the participants’ comments. Nevertheless, we received suggestions to improve its iPhone version, which was expected since this version of the app was functionally stable, but its user interface was less mature than the Android version. For instance, among the positive comments about the mobile apps, patients said: “The app was a great vehicle to gather that information, with the least inconvenience to the patient. My complements,” “easy to use and it does not take much time,” “I like the questions, they are simple, direct and give key elements to how I feel about things.” They also made comments about aspects that did not work so well: “Application had bugs and crashed as the OS progressed. User interface was visually unclear, and notifications were inconsistent or misleading” and “the sliding scale was not so responsive, the bar was very small, and it was hard sometimes to move the toggle.”

Additionally, 5 patients and 2 support persons completed a subset of 10 questions from the User Burden Scale [48] at the study exit phase. Table 13 shows the results, suggesting that using the toolset in the study was almost never perceived as a burden. Nevertheless, as attested by 3 respondents, the participant experience could be improved to reduce interaction times.

**Table 13:** Participants’ evaluation of a subset of questions from the User Burden Scale (N=7) [48].

Question	Never	A little bit of time	Sometimes	Very often	All of the time
App demands high mental effort	7	—^a^	—	—	—
App distracts me from social life	7	—	—	—	—
I worry about the information shared	7	—	—	—	—
I worry about app’s privacy policies	7	—	—	—	—
App is hard to learn	6	1	—	—	—
App has negative effect on social life	6	1	—	—	—
I need assistance to use the app	6	1	—	—	—
I need to remember too much	5	2	—	—	—
I spend too much time in the app	4	1	1	1	—
It takes me too long to do what I want	3	2	1	1	—

^a^Not applicable.

### Data Privacy

Careful treatment of sensitive data is an important legal, operational, ethical, and human factor in any human participant study. In adherence to the approved protocol, patients and support persons received a comprehensive set of information about the data privacy policies in four ways: (1) orally, during face-to-face meetings with the researchers at the beginning of the studies; (2) printed, as part of the informed consent forms; (3) displayed upon request from a menu in the mobile app used during the studies; and (4) shared upon request over email during the study. As a result, in this study, neither patients nor support persons made any comments about data privacy considerations, which is considered very positive.

## Discussion

### Principal Findings

This section discusses the results, providing recommendations for future adopters of the EMA and PeerMA methods in clinical research or clinical practice. We argue that the PeerMA method indeed provides unique information streams that could add value as an input to certain medical decision-making processes. We show examples where support person assessments (ie, PeerMA), as well as physical activity data, can be used to inform health professionals about the actual state of a patient regarding important outcomes such as hope, sleep quality, fatigue, pain, and depression. This section discusses results regarding the in-time similarity and correlations of the patient and the support person’s frequent health assessments conducted in their daily living. We discuss 4 human factors that influence the acceptance of the PeerMA method.

To begin with, aim 1 of our study, we believe that the time-aligned visual representation of several data streams shown in Figures 2-13 is useful to assist health personnel during the recovery period of patients after a transplant. Likewise, for patients awaiting a transplant, having access to a combination of objective measurements, such as the number of steps per day, with subjective ones reported by the actual patients and their support persons during their daily routines, may allow health professionals to complement their clinical assessment of a patient’s disease progression during stages prior to an organ transplant. Our illustrations in this article are only examples of very basic visualizations potentially informative to medical personnel; besides, it is possible to augment such visual representations of data by making them interactive. For example, medical professionals could select specific data points in a chart and study a patient’s medical record near that time (eg, accessing recent lab exams, x-ray, or ultrasound imagery, among others) next to these less additional information streams coming from a patient and their support persons. In the literature, when support persons complete a questionnaire with information about a patient, they play the role of proxies [13,21,23,25,26]. According to a study from the International Society for Quality of Life Research, the most common type of use of proxies is for pair studies in which patients and support persons use the same questionnaire (oftentimes with wording change for proxy completion) [29]. Those studies usually collect patients’ and support persons’ assessments sporadically (once every few weeks or months). Consequently, one benefit of pairing EMA with PeerMA consists of capturing more frequent assessments about a particular aspect of a patient, usually via short questionnaires with one or only a few questions.

Regarding the similarity of the EMA and PeerMA assessments between patients and their support persons, we noticed that the error metrics MAE, mean squared error, and root-mean-squared error are relatively small (around 10%-15%) for dyads 1 and 2, meaning that their regression curves representing the various assessments (hope, sleep, fatigue, depression, and pain) have a similar shape. We found the error metrics to be higher for dyads 3 to 7, with some of them showing high similarity; for instance, dyad 3 showing good similarity for fatigue, depression, and pain, and dyads 4, 5, and 7 showing good similarity for sleep quality. On the other hand, aside from dyad 2, The *R*^2^ metric reflects low-quality models in almost all the cases. It is natural to expect deviations because the support person’s assessments are not intended to be as exact as the patient’s assessments, but instead, a close approximation built from subjective perceptions issued by the patient’s support person. There are many reasons to explain differences in assessments between a patient and his/her support person. One could be a time shift, meaning that some time elapsed from the moment the patient manifested a certain behavior or attitude to the moment the support person perceived such a change and was able to report it in a PeerMA. The type of relationship and the degree of spatio-temporal proximity between a patient and a support person may be an important factor favoring a support persons’ ability to better perceive the emotional states of a patient [16-18]. However, we also know that self-reports (including EMA) may be affected by known problems such as socially desirable responding when a person answers questions untruthfully to attain a desirable outcome or consciously misrepresenting his/her actual state [13]. In summary, looking at the similarity between a patient’s and a support person’s frequent assessments is informative when analyzed carefully, considering the patient’s history, personal profile, as well as the type of relationship with his/her support person when possible.

Although we presented correlation results between EMA and PeerMA values, we do not discuss their statistical significance because they derive from a small sample of patients. We mostly wanted to expose the results in detail to motivate the reader and to support the identified findings. Despite that limitation, as shown in Table 11, the majority (77%) of correlations between patients and their support person assessments are positive (46% high, 20% moderate, and 11% weak). It means that for some dyads, the information streams could be valuable for medical personnel studying aspects of disease progression in a patient. For instance, if enough confidence is gained from the support person’s assessments about a patient, medical personnel could feed in information from the support person during a given day when a patient is unable to self-report his/her own state. In some cases, the correlations were negative. This is worth studying as well because that could be related to something simple, such as a support person failing to perceive and report the real patient’s state. However, it may also be more critical, such as a patient intentionally misrepresenting his/her actual state.

Regarding the identified human factors in this study, we learned that it is particularly important to promote self-awareness and add value to all the participants as they use the tools of EMA and PeerMA. It is worth exploring ways for the mobile app to show selected, meaningful EMA or PeerMA information to all the patients and support persons during the study (a form of compensation known as “in-kind”). We believe doing so will raise engagement and motivation, especially if done in near real-time and linked to the clinical decision-making for that particular patient. The lack of motivation leads to boredom for the patient and the support person. This unintended effect has been studied by Taylor et al [51]; van Berkel et al [52]; Mehrotra et al [53]; and Pejovic and Musolesi [54], who investigate gamification techniques (eg, scoring points, winning prizes, solving puzzles, and others) to increase engagement and participants’ satisfaction during mobile subject studies. Making the mobile apps more user-informative and interactive may positively influence the acceptance of EMA/PeerMA-based studies. Some of the patients suggested the addition of more app features to enhance their experience, for example, “add more elements to assess such as more activities, calories burnt and weight,” “questions may not be clear—glossary of terms of apps would help, eg, what is pain vs discomfort,” “when I get into how well you are sleeping, the scale needs to be thicker, or I should be able to have an option of drop-down, to be expressed differently.” However, these must be clearly co-designed with the care team to enhance the patient’s self-management skills while assuring the validity of the collected data.

Another important aspect concerns the selection of support persons. Because we recruited the patients during their regular visits to the hospital, their support person was already present for each patient. All the support persons were spouses of the respective patients, which gave us confidence that they were close enough to the patient and were worthy to participate in this exploratory study. However, in general, it should be evident that to use the PeerMA method effectively, the involved support persons should meet specific inclusion criteria at the individual level, as well as criteria regarding his or her relationship with the patient. These criteria may vary based on the nature or main attributes of the phenomena under study. The following are examples of such criteria that are derived from our research. (1) The patient and support person must have a close relationship, that is, in frequent contact with the patient. The overall frequency will depend on the phenomena under study. For instance, to study rapidly oscillating states such as changes in affect, support persons should be in contact with the patient a few times within a day. However, 2 or 3 interactions per week may still be effective for the PeerMA assessment to explore traits such as anxiety. From the reviewed literature [16-18], we know that the social proximity of the relationship is essential (eg, spouse, partner, boyfriend, girlfriend, close friends, and so on) to obtain truthful and meaningful results from the PeerMAs. (2) Patients should trust the support person. During PeerMA-based studies, support persons may start to think about the patient more often than they used to. By doing so and answering PeerMAs, support persons may become more aware of the states being experienced by the patient (or presumably close to the real state). Because these states are private to the patient, PeerMA will be more effective when the patients have high trust in the support person, such that they both feel confident and natural during the study. (3) Support persons should be skilled to perform the task. To begin with, support persons need procedural skills, including the correct use of the smartphone to keep it charged and connected to the internet, answering the PeerMA prompts on time, and providing honest responses. Additionally, support persons need cognitive skills, including the ability to observe and perceive the states of the patient (ie, interpersonal sensitivity) projected in countless forms, such as verbal and nonverbal expressions, behavior changes, and others. (4) Support persons should be motivated to participate. Motivation is not sufficient to perform as a support person, but it is necessary for the success of PeerMA-based studies. When recruiting support persons, researchers should clearly explain the implications of their task, as well as the unique value they bring to these types of studies. However, it must also be clear that playing the role of a support person represents commitment. (5) Support persons should be available to issue assessments. The degree of availability depends on the phenomena under study. For instance, if the PeerMA prompts occur during the day, the support persons should be available to answer the prompts as close to the time they are triggered as possible. Availability is essential to increase the ecological validity and significance of the PeerMA, which increases when they are answered within the same time window as when the EMA is responded to by the patient.

Finally, one may wonder about the optimal number of support persons to invite for each patient. In this study, we experimented only with one support person per patient since, in our sample, it was not possible to recruit 2 or more support persons for each patient. Having more than one support person would favor the collection of more observations, which has the potential to enhance the analyses; however, researchers should define this number based on the support persons’ alignment with the inclusion criteria of each study.

### Limitations

The first limitation of this work is the small population sample of 8 patients and their support persons. In our study, the support person of each patient always turned out to be the patient’s spouse, which is beneficial as we know the support person is close enough to the patient, but it also limits the generalizability of the results in our study. A second important limitation is the fact that part of the study occurred during the early months of 2020, and the patient-support person dynamics may have been altered by the world pandemic due to the COVID-19 virus. Additionally, study participants (both patients and their support persons) were recruited from only one location, the Liver Transplant Clinic at Stanford University Hospital. Consequently, the results may be biased towards this particular group of participants. We also consider as a limitation the absence of ground truth about the state of the patients and support persons when they answer EMAs and PeerMAs about the patients’ states, like fatigue [37], depression [38], hope [35], pain [39], and quality of sleep [36] during the study. Although the main goal of the studies was not to assess the reliability or agreement between the self-reported states (via EMA) and the actual state of the patient (ground truth), the lack of an objective measurement of the patient’s states affects the analysis of the peer-reported assessments (via PeerMA). Likewise, a related limitation is the lack of information about the physical and mental state of the support person when they issue assessments about the patient. We know that the state of the support persons, including their perceptions and personal beliefs, may bias how they perceive and report particular states occurring to the patient [55]. Another limitation is the lack of baseline or historical EMAs from the patients before they started the protocol, that is, before being “observed” by a support person [56]. The social dynamics between patients and support persons (even if they are family or close acquaintances) may induce a change in the typical behavior of the patient once they know that their support person is assessing them via PeerMA.

### Conclusions

In conclusion, we presented the results of a study implying a new, noninvasive data collection method named PeerMA to complement the well-known method of EMA. The motivation came from the limitation that patients are sometimes unable to provide reliable self-assessments of their mental or physical states (eg, due to the complexity of introspection and limitations due to health status, such as pre- or post-operatory, among others). In those cases, support persons could fill in the gaps in assessments and provide their own assessments about the patient based on the information they can perceive from them. In this pioneering study, we explored the PeerMA method in the context of real-life phenomena, collecting qualitative and quantitative data to assess the value of the method and the reason for its application in clinical care. The study was conducted with patients waiting for or after undergoing a liver transplant. We found empirical evidence about the feasibility of PeerMA as a method and showed that it provides unique information streams that are not available in traditional EMA-based studies. More precisely, we showed examples where support person assessments, as well as wearable-based physical activity data, can be used to inform health professionals about the actual state of a patient regarding important health outcomes such as hope, sleep quality, fatigue, pain, and depression. We identified and discussed 4 human factors that influence the acceptance of the PeerMA method. Overall, we show that it is possible to leverage the data acquired via the PeerMA method as well as wearable activity datasets as complementary to EMA to better study and understand the disease recovery pathways of high-risk populations, such as patients recovering from an organ transplant.

### Future Work

Because this is the first study using the PeerMA method in patient populations, we identified several avenues that are worth exploring in future studies to obtain more information about the strengths and weaknesses of the method. The first one implies a type of new interaction where we ask the support person to only “validate” the EMA scores issued by the patient. In this scenario, patients would respond to the EMA (as usual), and at that point, a PeerMA would be sent to the support person. But, instead of asking the support person to complete their independent PeerMA assessment, their task is to react upon it, for instance: (1) they agree/disagree with the EMA; (2) they suggest their own PeerMA assessment; or (3) they opt not to respond. Related to that, we think it is important to validate the implications of using rating scales that were not designed for observer assessments; those rating scales were originally designed for self-assessments [29]. Similarly, there is a need to validate the implications, in both self and observer assessments, of using rating scales for frequently repeated measures when those rating scales were originally designed to be applied just one time, or only very infrequently. It is important to define the frequency of the assessments that researchers use in EMA-based studies, considering the frequency at which the evaluated human states naturally oscillate in the sampled population of patients (eg, hope, fatigue, depression, and pain, among other states).

A second aspect to explore concerns the roles of the participants in the studies. PeerMA-based studies imply the actions of a social group, that is, a patient and one or more support persons. This study used only unidirectional interactions, and both the patient and the support person issued assessments about the patient, which indirectly enabled social support to be provided to the patient by the support person. Future work will study the possible benefits of bidirectional interactions; when the cognitive load of the patient allows it, participants could take both roles of patient and support person simultaneously; that way, each person completes EMAs about their own state and PeerMAs about the other person. This variation may have positive side effects, such as increasing engagement by virtue of the implied mutual support. However, it may also make it difficult to design the study to ensure the validity of the collected data.

Another aspect to explore is the degree of expressiveness embedded in EMA and PeerMA questions. This study used only validated numeric scales, which limits how much one can assess one’s own emotions, feelings, or mental or physical state. We would like to explore other assessment alternatives for patients and support persons. The first one is to openly choose the moods, states, or behaviors they wish to report at a given moment, instead of having to assess predefined states over and over. The possible states could be picked from a predefined list to preserve consistency across patients and reduce patient burden. This may have positive effects in cases where individuals feel the monotony of regularly assessing the same fixed states. However, such a patient-driven EMA/PeerMA data collection design may induce bias stemming from collecting only the states that patients and support persons want to report, which may or may not be sufficiently valid or relevant to the study goals or to be used for clinical decision making. Another alternative is to allow participants to describe the current state/behaviors rather than just producing ratings on a scale. This could be beneficial for certain patients to do introspection of their states/behaviors, for example, those who are at risk of relapses from certain addictions or behaviors they wish to change. Instead of typing in text, they could record audio describing their current state. A third alternative is to allow patients to define the states/behaviors they want their support persons to assess. Patients could define (eg, by choosing from a predefined list, or by proposing) the state or construct that they want (and feel comfortable with) their support persons to assess. Possible advantages include the reduction of bias of being observed [56] and emerging knowledge, such as understanding the natural emergence of states that patients allow their support persons to assess (rather than being imposed upon them). Nevertheless, a direct disadvantage is that this type of design may not be directly valuable for clinical researchers who are interested in studying a particular human state that may have clinical, health outcomes, and quality of care implications, or may not be directly valuable for clinical decision-making.

We also would like to study the effects of patients’ and support persons’ proximity when they participate in EMA/PeerMA studies. The general stance is that being often physically close to the patient adds higher confidence to the support person’s reports; however, this may not be guaranteed, as certain human states are hard to detect immediately, even when in front of the individual experiencing that state. In addition, the ability to detect someone's state varies among individuals. As such, not being physically close to the patient or not having had a recent face-to-face encounter does not preclude support persons from reporting with high confidence. Nowadays, individuals can communicate easily via several channels, leveraging a variety of smartphone messaging apps and social networking apps, which may provide enough cues for support persons to confidently infer the state of a given person they are acquainted with. In our study, the EMAs and PeerMAs were triggered based on an interval contingent trigger [57] (ie, using fixed time scheduling), which does not discriminate among physical proximity states. Physical proximity between patients and support persons could be inferred using technologies such as Bluetooth, WiFi signals, and Geo-fences, among other methods to infer a person’s location. Identification of digital proximity, on the other hand, could leverage usage patterns of social network apps and communication exchanges via SMS and phone calls, among others. A less sophisticated but sufficient method could be asking the support person directly, “Is the patient next to you to answer a short survey?” or “Have you recently been in contact with the patient to answer a short survey?” If the answer is no, the support person could still decide if he/she wants to answer the PeerMA survey and provide a confidence level based on his/her knowledge of the target person. Linking EMA and PeerMA surveys to physical or digital proximity may help reduce the burden on patients and support persons and ensure higher data quality.

Additionally, in this study, support persons were able to express their confidence level for each individual PeerMA answer. They may have selected low confidence if they had not been in contact (either physical or digital) with the patient in the recent past. However, our design asked the question of confidence at the end of each PeerMA, which forced the support person to complete the surveys, even in cases where they had low confidence. An alternative approach can be that, after a few days (when the support persons become familiar with the types of questions in the surveys), the question of confidence appears at the beginning of the survey, and then, based on the first response, an algorithmic decision is made to present, or not, the actual PeerMA questions. This may reduce the burden, especially on the support person’s side.

## Abbreviations

EMA: ecological momentary assessment
MAE: mean absolute error
PeerMA: peer-ceived momentary assessment

## Data Availability

The data sets generated and analyzed during this study are not publicly available because the data usage authorized by study participants via informed consent was restricted to the study researchers. Please refer to the corresponding author for reasonable data requests.
